# Endoscopic endonasal transcavernous approach for invasive corticotroph pituitary adenoma

**DOI:** 10.3171/2020.4.FocusVid.19949

**Published:** 2020-04-01

**Authors:** Ming-Ying Lan, Wei-Hsin Wang

**Affiliations:** Departments of 1 Otorhinolaryngology–Head and Neck Surgery and; 2 Neurosurgery, Taipei Veterans General Hospital, Taipei; and; 3 School of Medicine, National Yang-Ming University, Taipei, Taiwan

**Keywords:** endonasal endoscopic approach, transcavernous approach, pituitary adenoma, Cushing’s disease, surgical video

## Abstract

This is a 37-year-old woman who presented with weight gain, a moon-shaped face, and muscle weakness for 4 months. Cushing’s disease was confirmed after a series of diagnostic tests. MRI demonstrated a pituitary macroadenoma with right cavernous sinus invasion and encasement of the right ICA. An endoscopic endonasal approach was performed, and gross-total resection could be achieved without injury of the cranial nerves. The Cushing’s syndrome improved gradually after the surgery. Histopathology revealed a corticotroph adenoma. In this surgical video, we demonstrate the strategies of tumor resection according to a surgical anatomy-based classification of the cavernous sinus from an endonasal perspective.

The video can be found here: https://youtu.be/aNXFRdGfjpI.

## Transcript

This is a case of an endoscopic endonasal approach for invasive corticotroph pituitary adenoma. This 37-year-old woman presents with typical Cushing’s syndrome, and the diagnostic tests show compatible with Cushing’s disease. Here you can see the tumor occupy the whole sphenoid sinus with invasion into right cavernous sinus. Right ICA is completely encased **(0:29)**. Here the animation **(0:39)** shows the anatomy of cavernous sinus and relationship of lesions to the surrounding neurovascular structures. The tumor is very invasive and occupies all compartments of right-side cavernous sinus. Here we can see the intraoperative positioning **(0:53)**. Partial resection of right middle turbinate and ethmoidectomy **(0:57)** are performed for better visualization and exposure. The rescued flap **(1:06)** is performed on left side followed by posterior nasal septectomy. Next shows right pterygopalatine fossa is exposed **(1:16)**. The tumor in left sphenoid sinus can be removed by careful peeling. The Doppler is used to localize right clinoid segment of ICA **(1:25)**. Right lateral recess of sphenoid sinus is occupied by the tumor **(1:33)**. Partial transpterygoid approach is needed for tumor removal. Here we can see right vidian nerve **(1:38)**. The palatovaginal artery which leads to vidian nerve **(1:45)** is controlled and cut to allow mobilization of structures inside the pterygopalatine fossa. Here we can see the vesalius vein **(1:55)**, which connects the cavernous sinus and the pterygoid plexus. Severe oozing may be encountered while removing tumor in the depth of vidian canal. Here we can identify the maxillary strut that separates the V2 from the V1 (or superior orbital fissure). The maxillary strut is a constant landmark to localize the anterior border of the lateral compartment of cavernous sinus **(2:12)**. The feather knife is used to open the dura anteriorly **(2:15)**. Care should be taken for prevention of avulsion injury from cavernous ICA. Two-suctions technique is efficient to remove the soft tumor. Now we proceed to the lateral compartment of cavernous sinus **(2:28)**. It is always challenging to mobilize the ICA medially. The abducens nerve should be identified early on to prevent injury during tumor resection **(2:45)**. The other cranial nerves in the lateral compartment include oculomotor nerve, trochlear nerve, and V1. The branches of inferior lateral trunk need to be carefully identified and controlled. Here the inferior compartment is exposed, which represents the space anterior to the vertical segment of cavernous ICA. Sympathetic plexus can be identified here, which usually conjoint with abducens nerve laterally **(3:08)**. Next, we will proceed to superior (or medial) compartment of cavernous sinus. Here we dissect out the medial wall of cavernous sinus from the ICA **(3:19)**. Careful resecting the medial wall and the ligaments attached on it can further mobilize ICA laterally and completely expose the oculomotor triangle. Here we can see the interclinoid ligament and the oculomotor nerve can be identified laterally **(3:45)**. Here we can see the dura defect, and this is the site where tumors extending into the posterior fossa **(3:50)**. Next the case proceeds to the posterior compartment of cavernous sinus. Posterior compartment is the space just behind the vertical segment of cavernous ICA. After penetrating out two layers of dura, the abducens nerve can be identified at the bottom of posterior compartment **(4:09)**. Since right ICA and cavernous sinus are exposed, a rescued nasal septal flap is harvested and covered on the dura defect. The free middle turbinate flap is covered on the debrided septum. Here we can see the postop result with complete treatment. The patient’s postop cranial nerves functions are well preserved without any deficit and she is under remission status now.
